# Associations of the systolic and diastolic components of orthostatic hypotension with markers of cardiovascular risk in older men: A cross‐sectional analysis from The British Regional Heart Study

**DOI:** 10.1111/jch.13996

**Published:** 2020-08-16

**Authors:** Artaza Gilani, Sheena E. Ramsay, Stephen P. Juraschek, Olia Papacosta, Lucy T. Lennon, Peter H. Whincup, Sasiwarang Goya Wannamethee

**Affiliations:** ^1^ Research Department of Primary Care & Population Health Royal Free Campus University College London London UK; ^2^ Population Health Sciences Institute Newcastle University Newcastle upon Tyne UK; ^3^ Division of General Medicine Section for Research Beth Israel Deaconess Medical Center Harvard Medical School Boston MA USA; ^4^ Population Health Research Institute, St George's University of London London UK

**Keywords:** cardiovascular disease, cardiovascular disease risk factors, epidemiology, hypertension, orthostatic hypotension

## Abstract

The mechanisms underlying the association between orthostatic hypotension (OH) and cardiovascular disease are unclear. We investigated whether OH is associated with circulating cardiovascular risk markers. This was a cross‐sectional analysis of 3857 older, community‐dwelling men. “Consensus OH” was defined as a sitting‐to‐standing decrease in systolic blood pressure ≥20 mm Hg and/or diastolic blood pressure ≥10 mm Hg that occurred within three minutes of standing. Multiple generalized linear regression and logistic models were used to examine the association between cardiovascular risk markers and OH. Consensus OH was present in 20.2%, consisting of isolated systolic OH in 12.6%, isolated diastolic OH in 4.6%, and combined systolic and diastolic OH in 3.0%. Concentration of von Willebrand factor, a marker of endothelial dysfunction, was positively associated with isolated systolic OH (OR 1.35, 95% CI 1.05‐1.73) and combined systolic and diastolic OH (OR 2.27, 95% CI 1.35‐3.83); high circulating phosphate concentration, which may reflect vascular calcification, was associated with isolated diastolic OH (OR 1.53, 95% CI 1.04‐2.25) and combined systolic and diastolic OH (OR 2.12, 95% CI 1.31‐3.44), high‐sensitivity troponin T, a marker of myocardial injury, was positively associated with isolated diastolic OH (OR 1.69, 95% CI 1.07‐2.65) and N‐terminal pro‐brain natriuretic peptide, a marker of cardiac stress, was positively associated with combined systolic and diastolic OH (OR 2.14, 95% CI 1.14‐4.03). In conclusion, OH is associated with some cardiovascular risk markers implicated in endothelial dysfunction, vascular calcification, myocardial injury, and cardiac stress. Clinicians should consider assessing cardiovascular risk in patients with OH.

## 1 INTRODUCTION

Orthostatic hypotension (OH) is an age‐dependent physical sign^1^ that is present in almost one in five community‐dwelling adults who are aged 60 years or above.^2^ Mounting observational data have emerged over the last two decades suggesting OH increases risk of all‐cause mortality,^3^, ^4^ and that it may be an independent risk factor for cardiovascular disease, including stroke, heart failure, atrial fibrillation, and myocardial infarction.^4^, ^5^, ^6^, ^7^, ^8^ Indeed, OH has been associated with subclinical atherosclerotic vascular disease^9^ and structural and functional cardiac changes, including left ventricular hypertrophy and diastolic dysfunction.^10^ However, there is no consensus regarding the underlying mechanisms which explain how OH results in these pathological changes and increases cardiovascular risk.

There are limited data examining the association between circulating cardiovascular risk markers and OH. A small, cross‐sectional analysis has shown OH to be associated with the inflammatory biomarkers midkine, immunoglobulin‐like transcript 3, and regenerating islet‐derived protein 4.^11^ Another small, cross‐sectional analysis has shown OH to be associated with increased concentration of von Willebrand factor (VWF),^12^ a glycoprotein involved in hemostasis that is implicated in both endothelial dysfunction and activation.^13^ In a large, prospective analysis, OH was associated with higher levels of high‐sensitivity troponin T (hs‐troponin T), a marker of myocardial injury, and N‐terminal pro‐brain natriuretic peptide (NT‐proBNP), a marker of cardiac stress.^9^ These findings implicate inflammation and endothelial dysfunction—both important contributors to atherosclerosis,^14^, ^15^ a major cause of cardiovascular disease—as well as myocardial injury and cardiac stress as mechanisms that may explain the association between OH and increased risk of cardiovascular disease.

However, there are specific limitations to these studies. Firstly, the study showing an association between inflammatory biomarkers and OH was in a cohort of less than 300 people, while the study showing an association between VWF and OH was in less than 200 people. Secondly, the study showing an association between OH and hs‐troponin T and NT‐proBNP was done in middle‐aged adults, in whom OH is approximately five to six times less prevalent than in older adults.^1^, ^5^ Therefore, to further understand how OH increases risk of cardiovascular disease in older adults, in whom the burden of OH is greatest, we examined the association between OH and circulating cardiovascular risk markers implicated in metabolic risk, inflammation, endothelial dysfunction, vascular calcification, myocardial injury, and cardiac stress.

## 2 METHODS

### 2.1 Study population

Study participants were from The British Regional Heart Study, which is an ongoing prospective cohort study that initially recruited 7735 men aged 40‐59 from one general practice in each of 24 British towns between 1978 and 1980. The sample was socioeconomically representative of the population. Participants were predominantly (>99%) of white European ethnicity. Sampling methods have been described previously.^16^ Ethical approval was obtained from the National Research Ethics Service Committee London.

For the 20th year of follow‐up, all surviving men were invited for re‐examination, which took place between 1998 and 2000. The re‐examination involved a self‐completed questionnaire, about lifestyle and medical history, undergoing physical examination and venepuncture. There were 4252 men who completed the questionnaire and underwent physical examination (77% response rate). Anthropometric and physiological measurements included height, weight, waist and hip circumference, sitting and standing blood pressure, heart rate, forced expiratory volume in one second, and forced vital capacity. Specific methods used for data collection have been described previously.^16^, ^17^

Of the 4252 men, 4045 had measurements of biochemistry. From this sample, 117 men with prevalent heart failure were excluded because heart failure is associated with hypertension,^18^ which itself is a major determinant of OH,^6^, ^19^ and patients with heart failure have exceptionally high risk of cardiovascular and all‐cause mortality.^20^ Of the remainder, 71 participants had incomplete sitting and standing blood pressure measurements. They were also excluded, leaving 3857 participants for the purpose of this analysis.

### 2.2 Blood pressure measurements

Sitting and standing blood pressure measurements were taken on the right arm using an automatic Dinamap 1846SX. The Dinamap blood pressure monitor overestimated systolic blood pressure by ~8 mm Hg compared with the standard mercury sphygmomanometer which was the standard reference instrument for blood pressure measurement at the time.^21^ 8 mm Hg was therefore subtracted from the raw systolic blood pressure readings. The cuff was sized according to arm circumference: between 28 cm and 35 cm and a standard adult cuff was used, <28 cm and a small adult cuff was used and >35 cm and a large adult cuff was used. The bladder center of the cuff was aligned over the brachial artery. Participants were asked to rest their arm on the examination table, such that the upper arm was at chest level, for sitting measurements.

The Dinamap was set to take repeated measurements at one minute intervals. Four consecutive blood pressure measurements were taken: two sitting followed by two standing. Participants had not been seated nor supine for a prescribed duration prior to the first measurement. The participant was asked to stand after the second sitting measurement was completed. The first standing blood pressure measurement began within one minute of the participant standing, while the second standing blood pressure measurement began after one minute of standing. The interval between the start of the first sitting measurement and the completion of the second standing measurement was 3.5 minutes.

### 2.3 Definition of OH

“Consensus OH” was defined as a decrease in either systolic blood pressure ≥20 mm Hg or diastolic blood pressure ≥10 mm Hg, or both, that occurred between either the first or second standing blood pressure measurements and the mean of the two sitting blood pressure measurements. These specific decreases in blood pressure on standing to define OH are endorsed by the American Autonomic Society, the European Federation of Autonomic Societies, the Autonomic Research Group of the World Federation of Neurology, and the Autonomic Disorders section of the American Academy of Neurology and described in their consensus statement.^22^ In addition, a second classification distinguished subtypes within this group. If the decrease only affected systolic blood pressure, this was classified as “isolated systolic OH.” If the decrease only affected diastolic blood pressure, this was classified as “isolated diastolic OH.” If the decrease affected both systolic and diastolic blood pressure, this was classified as “combined systolic and diastolic OH.” We examined the subtypes comprising consensus OH because of evidence suggesting the individual components of OH are of different prognostic value: The systolic component appears more predictive of stroke and the diastolic component more predictive of myocardial infarction, for example.^23^

### 2.4 Measurement of blood markers

At the physical examination, a fasting blood sample was taken for laboratory measurements of hematology and biochemistry, including total cholesterol, low‐density lipoprotein (LDL), high‐density lipoprotein (HDL), triglycerides, glucose, urate, phosphate, fibrinogen, von Willebrand Factor (VWF), D‐dimer, c‐reactive protein (CRP), interleukin‐6 (IL‐6), high‐sensitivity troponin T (hs‐troponin T), and N‐terminal pro‐brain natriuretic peptide (NT‐proBNP). We chose to examine these markers in this study because they are implicated in cardiometabolic risk, hemostatis and endothelial dysfunction, vascular calcification, myocardial injury, and cardiac stress, mechanisms that may, in part, explain the association between OH and cardiovascular outcomes.^9^, ^11^, ^12^ Two extreme outliers—an NT‐proBNP of 35 000 pg/mL and hs‐troponin T of 1301 pg/mL—were set to missing. Details of specific measurement methods have been described previously.^24^, ^25^, ^26^, ^27^, ^28^, ^29^, ^30^, ^31^

### 2.5 Statistical methods

Statistical analyses were performed using SAS 9.4. Continuous variables that were skewed were log‐transformed to approximate normality for parametric tests. Skewed variables were CRP, IL‐6, D‐dimer, fibrinogen, glucose, triglycerides, NT‐proBNP, and hs‐troponin T. Hypothesis testing was first applied to mean unadjusted biomarker values in univariate generalized linear regression analyses. Where statistically significant associations, as per Bonferroni adjustment (*P* < .05/14 tested markers), arose from this analysis, these biomarkers were selected for further analyses in both multiple generalized linear and logistic models. For the logistic analyses, the biomarkers (independent variable) were grouped into tertiles to examine whether elevated levels (top tertile vs bottom tertile) were associated with increased odds of OH (dependent variable).

The multiple linear, and logistic, regression models were adjusted for age, body mass index, mean sitting systolic blood pressure, resting pulse, total cholesterol, smoking status (never smoked, ex‐smoker for 0‐15 years, ex‐smoker for >15 years, and current smoker), alcohol use (0‐15 units per week and >16 units per week), physical activity (inactive or not inactive), social class (manual or non‐manual) and the presence of prevalent stroke, myocardial infarction, atrial fibrillation, diabetes, chronic kidney disease (CKD), chronic obstructive pulmonary disease (COPD), and current antihypertensive medication use. CKD was defined as an estimated glomerular filtration rate (eGFR) <60 mL/min/1.73 m^2^, as estimated from serum creatinine using the Modification of Diet in Renal Disease equation.^32^ Hypertension was defined as mean sitting systolic blood pressure ≥140 mm Hg and/or mean sitting diastolic blood pressure ≥90 mm Hg (as per the 2018 European Society of Cardiology‐European Society of Hypertension and 2019 National Institute for Health and Care Excellence guidelines^33^) and/or antihypertensive medication use. Antihypertensive medications were defined as use of any antihypertensive medication as per British National Formulary (version 38) code 3.1. COPD was defined as a forced expiratory volume in one second to forced vital capacity ratio of <0.7. Atrial fibrillation was defined according to Minnesota codes 8.3.1 and 8.3.3.

Case definitions were obtained from primary care record reviews and have been reported previously.^17^ Methods of measurement and classification for measures of lipids, lung function, smoking status, physical activity, alcohol intake, and social class have also been reported previously.^34^, ^35^

A sensitivity analysis was done by excluding participants with prevalent stroke and myocardial infarction (n = 352).

## 3 RESULTS

### 3.1 Prevalence and baseline characteristics

Of the 3857 participants (mean age 68.6 years, standard deviation 5.5 years), 778 men (20.2%) had consensus OH. Among these, 485 (12.6%) had isolated systolic OH, 177 (4.6%) had isolated diastolic OH, and 116 (3.0%) had both systolic and diastolic OH. Only 10.3% of participants with consensus OH were breathless or felt faint within three minutes of standing.

Consensus OH was positively associated with age, sitting systolic blood pressure, sitting diastolic blood pressure, heart rate, and resting pulse ≥90 beats per minute. It was not associated with smoking status or alcohol consumption. There were differences in the distribution of baseline characteristics across the components of OH: Heart rate was associated with isolated diastolic OH, but not isolated systolic OH, while BMI was associated with isolated systolic OH, but not isolated diastolic OH, for example (Table 1).

**Table 1:** Baseline characteristics of the study population

	No OH (n = 3079)	COH (n = 778)	*P*	ISOH (n = 485)	*P*	IDOH (n = 177)	*P*	SDOH (n = 116)	*P*
	Mean (SD)	Mean (SD)		Mean (SD)		Mean (SD)		Mean (SD)	
Age, years	68.4 (5.47)	69.5 (5.50)	<.0001	69.4 (5.39)	.0002	69.4 (5.65)	.0229	70.0 (5.78)	.0016
Body mass index, kg/m^2^	26.9 (3.61)	26.6 (3.68)	.0527	26.2 (3.58)	<.0001	27.4 (3.78)	.0609	27.2 (3.72)	.3325
Waist Circumference, cm	97.0 (10.23)	97.0 (10.56)	.9652	95.8 (10.45)	.0182	99.0 (10.23)	.0108	98.8 (10.89)	.0737
Sitting systolic blood pressure, mm Hg	146.6 (23.45)	160.6 (23.90)	<.0001	159.1 (22.84)	<.0001	159.0 (22.36)	<.0001	169.6 (28.35)	<.0001
Sitting diastolic blood pressure, mm Hg	84.3 (10.70)	89.4 (12.17)	<.0001	86.3 (11.26)	.0002	93.8 (11.55)	<.0001	95.3 (12.61)	<.0001
Heart rate^a^, beats per minute	65 (11.68)	66 (12.49)	.0045	66 (13.31)	.3705	67 (13.78)	.0380	69 (13.50)	.0003
Arm circumference, cm	30.4 (2.77)	30.0 (2.76)	.0001	29.9 (2.69)	<.0001	30.2 (2.90)	.2211	30.3 (2.82)	.6191

	%	%	*P*	%	*P*	%	*P*	%	*P*
Current smokers	12.70	14.00	.3404	15.26	0.1245	7.91	.0622	18.10	.0921
Moderate to heavy alcohol consumption	19.89	18.85	.5183	18.70	0.5441	20.69	.7968	16.67	.3976
Physically inactive	32.86	37.05	.0308	35.06	0.3497	38.73	.112	42.73	.0320
Manual Social Class	50.50	52.45	.3323	51.55	0.6683	59.43	.0222	45.69	.3093
Resting tachycardia^a^ (heart rate ≥ 90 beats per minute)	3.13	5.39	.0034	5.49	0.0100	5.00	.1956	5.56	.1659
Breathless or faint when standing	8.41	10.28	.1001	8.87	0.7386	12.99	.0367	12.07	.1693

In all cases, the reference group for hypothesis testing was “No OH.”

Abbreviations: COH, consensus orthostatic hypotension; IDOH, isolated diastolic orthostatic hypotension; ISOH, isolated systolic orthostatic hypotension; SDOH, combined systolic and diastolic orthostatic hypotension.

^a^ These measures exclude 132 men with prevalent atrial fibrillation.

### 3.2 Co‐morbid conditions

Consensus OH was associated with increased odds of prevalent CKD, COPD, antihypertensive medication use, and hypertension (Table 2). The co‐morbidities were differentially distributed among the components of OH. COPD was statistically significantly associated with isolated systolic OH, but not isolated diastolic OH, while atrial fibrillation was associated with isolated diastolic OH, but not isolated systolic OH. With the exception of COPD and CKD, the co‐morbidities appeared to cluster with the diastolic component of OH.

**Table 2:** Co‐morbidities of study participants stratified by OH status

Co‐morbidity	No OH	COH	*P*	ISOH	IDOH	SDOH	COH	ISOH	IDOH	SDOH
	%	%		%	%	%	Odds ratio (95% Confidence interval)			
Myocardial Infarction	6.82	5.66	.2426	4.74	8.47	5.17	0.82 (0.59‐1.15)	0.68 (0.44‐1.06)	1.27 (0.73‐2.19)	0.75 (0.32‐1.72)
Stroke	2.83	3.34	.4459	2.47	5.08	4.31	1.19 (0.76‐1.86)	0.87 (0.47‐1.61)	1.84 (0.91‐3.72)	1.55 (0.62‐3.89)
Atrial Fibrillation	3.19	4.38	.1044	2.07	9.09	6.90	1.39 (0.93‐2.07)	0.64 (0.33‐1.24)	3.04 (1.75‐5.27)	2.25 (1.07‐4.74)
Diabetes mellitus	5.75	6.04	.7553	4.12	9.04	9.48	1.05 (0.76‐1.47)	0.71 (0.44‐1.13)	1.63 (0.95‐2.78)	1.72 (0.91‐3.26)
Chronic kidney disease	13.80	16.90	.0281	16.12	15.91	21.74	1.27 (1.03‐1.57)	1.20 (0.92‐1.56)	1.18 (0.78‐1.79)	1.74 (1.10‐2.74)
Antihypertensive medication use	30.02	35.09	.0064	35.46	33.33	36.21	1.26 (1.07‐1.49)	1.28 (1.05‐1.57)	1.17 (0.85‐1.61)	1.32 (0.90‐1.95)
Chronic obstructive pulmonary disease	25.10	31.91	.0001	33.06	28.00	33.04	1.4 (1.18‐1.66)	1.47 (1.20‐1.81)	1.16 (0.83‐1.63)	1.47 (0.99‐2.19)
Hypertension	72.43	87.15	<.0001	84.95	90.40	91.38	2.58 (2.06‐3.23)	2.15 (1.66‐2.79)	3.58 (2.16‐5.94)	4.04 (2.10‐7.76)

Abbreviations: COH, consensus orthostatic hypotension (n = 778); IDOH—isolated diastolic orthostatic hypotension (n = 177); ISOH, isolated systolic orthostatic hypotension (n = 485); No OH (n = 3079); SDOH—combined systolic and diastolic orthostatic hypotension (n = 116).

The odds ratios are unadjusted and compare the odds of co‐morbidity among participants with OH, or its components, to those without OH.

### 3.3 Blood markers

In univariate linear regression analyses, consensus OH was associated with higher levels of phosphate, IL‐6, VWF, D‐dimer, NT‐proBNP, and hs‐troponin T. Total cholesterol, LDL, HDL, triglycerides, glucose, urate, fibrinogen, and CRP were not statistically significantly associated with consensus OH (Table 3). The markers which were statistically significantly associated with OH were selected for multiple generalized linear regression, and logistic regression, analyses in relation to the systolic and diastolic components of OH. In the multiple linear regression analyses (Table 4), VWF was associated with isolated systolic OH; phosphate was associated with isolated diastolic OH, and combined systolic and diastolic OH and NT‐proBNP was associated with combined systolic and diastolic OH. These associations remained robust in a sensitivity analysis, in which participants with prevalent myocardial infarction and stroke were excluded (Table 5).

**Table 3:** Univariate linear regression analysis of the association between cardiovascular risk markers and consensus OH. The values presented are means with their standard error

	No OH (n = 3079) Mean (SE)	COH (n = 778) Mean (SE)	*P*
Metabolic markers			
Cholesterol, mmol/L	6.00 (0.02)	6.02 (0.04)	.7682
LDL, mmol/L	3.88 (0.02)	3.89 (0.04)	.6866
HDL, mmol/L	1.31 (0.01)	1.35 (0.01)	.0074
Triglycerides,^a^ mmol/L	1.63 (1.01)	1.57 (1.02)	.0919
Glucose,^a^ mmol/L	5.87 (1.00)	5.93 (1.01)	.2009
Urate, mmol/L	0.38 (<0.01)	0.39 (<0.01)	.3278
Inflammatory markers			
CRP,^a^ mg/L	1.70 (1.02)	1.77 (1.04)	.3361
IL‐6,^a^ pg/mL	2.39 (1.01)	2.59 (1.02)	.0023^*^
Markers of hemostatis			
Fibrinogen,^a^ g/L	3.16 (1.00)	3.22 (1.01)	.0241
VWF, IU/dL	137.07 (0.81)	147.04 (1.74)	<.0001^*^
D‐dimer,^a^ ng/mL	81.45 (1.01)	91.84 (1.03)	.0004^*^
Marker of mineral metabolism			
Phosphate, mmol/L	1.15 (<0.01)	1.17 (0.01)	.0013^*^
Markers of cardiac stress and myocardial injury			
NT‐proBNP,^a^ pg/mL	91.84 (1.02)	117.92 (1.04)	<.0001^*^
Hs‐troponin T,^a^ pg/mL	11.7 (1.01)	12.68 (1.02)	<.0001^*^

The number of missing values for each variable out of sample size of 3857 was as follows: Cholesterol 12, LDL 67, HDL 35, triglycerides 11, glucose 13, urate 9, phosphate 31, CRP 31, IL‐6 34, fibrinogen 8, VWF 6, D‐dimer 9, NT‐proBNP 248, hs‐troponin T 25.

^a^ Geometric mean (standard error); all other values are the arithmetic mean (standard error).

* Statistically significant after Bonferroni adjustment (*P* < .05/14 tested markers, ie, *P* < .0036).

**Table 4:** Multiple linear regression analysis of the association between cardiovascular risk markers, OH and its individual components. The values presented are means with their standard error

Blood marker	No OH (n = 3079)	COH (n = 778)	*P*	ISOH (n = 485)	*P*	IDOH (n = 177)	*P*	SDOH (n = 116)	*P*
	Mean (SE)	Mean (SE)		Mean (SE)		Mean (SE)		Mean (SE)	
IL‐6,^*^, ^a^ pg/mL	2.40 (1.01)	2.47 (1.02)	.2983	2.40 (1.03)	.9916	2.49 (1.05)	.4406	2.74 (1.06)	.0292
Phosphate, mmol/L	1.15 (<0.01)	1.18 (0.01)	<.0001^*^	1.16 (0.01)	.0988	1.19 (0.01)	.0011^*^	1.21 (0.01)	.0001^*^
VWF, IU/dL	137.38 (0.80)	145.36 (1.64)	<.0001^*^	144.70 (2.05)	.0010^*^	145.46 (3.39)	.0207	148.09 (4.18)	.0122
D‐dimer,^*^, ^a^ ng/mL	82.73 (1.01)	86.70 (1.03)	.1499	83.92 (1.04)	.7102	85.15 (1.06)	.6362	102.82 (1.08)	.0038
NT‐proBNP,^*^, ^a^ pg/mL	94.85 (1.02)	101.00 (1.04)	.1285	91.86 (1.05)	.5219	108.95 (1.08)	.0828	136.09 (1.10)	.0001^*^
Hs‐troponin T,^*^, ^a^ pg/mL	11.72 (1.01)	12.12 (1.02)	.0692	12.00 (1.02)	.2983	12.42 (1.03)	.0983	12.21 (1.04)	.3399

Abbreviations: COH, consensus orthostatic hypotension; IDOH, isolated diastolic orthostatic hypotension; ISOH, isolated systolic orthostatic hypotension; SDOH, combined systolic and diastolic orthostatic hypotension.

The multiple regression models adjusted for age, body mass index, mean sitting systolic blood pressure, resting pulse, smoking status, alcohol consumption, social class, physical activity, presence of prevalent stroke, myocardial infarction, atrial fibrillation, diabetes mellitus, chronic kidney disease, chronic obstructive pulmonary disease, and current antihypertensive medication use. In all cases of hypothesis testing, “No OH” was the reference group.

^a^ Geometric mean (standard error); all other values are the arithmetic mean (standard error).

* Statistically significant after Bonferroni adjustment (*P* < .0036).

**Table 5:** Multiple linear regression sensitivity analysis of the association between cardiovascular risk markers, OH, and its individual components. The values presented are means with their standard error. The sensitivity analysis excluded 352 participants with prevalent stroke and/or myocardial infarction

Blood marker	No OH (n = 2795)	COH (n = 710)	*P*	ISOH (n = 450)	*P*	IDOH (n = 155)	*P*	SDOH (n = 105)	*P*
	Mean (SE)	Mean (SE)		Mean (SE)		Mean (SE)		Mean (SE)	
Phosphate, mmol/L	1.15 (<0.01)	1.18 (0.01)	<.0001^*^	1.17 (0.01)	.0341	1.19 (0.01)	.0057	1.21 (0.02)	.0002^*^
VWF, IU/dL	135.94 (0.84)	144.48 (1.70)	<.0001^*^	143.37 (2.11)	.0012^*^	146.07 (3.60)	.0062	147.00 (4.34)	.0128
D‐dimer,^a^ ng/mL	79.90 (1.01)	84.34 (1.03)	.1042	82.44 (1.04)	.4273	82.38 (1.06)	.632	96.75 (1.08)	.013
NT‐proBNP,^a^ pg/mL	87.99 (1.02)	92.36 (1.04)	.2568	84.94 (1.05)	.4909	98.64 (1.09)	.1745	121.99 (1.10)	.0009^*^

Abbreviations: COH, consensus orthostatic hypotension; IDOH, isolated diastolic orthostatic hypotension; ISOH, isolated systolic orthostatic hypotension; SDOH, combined systolic and diastolic orthostatic hypotension.

The multiple regression models adjusted for age, body mass index, mean sitting systolic blood pressure, resting pulse, smoking status, alcohol consumption, social class, physical activity, presence of prevalent atrial fibrillation, diabetes mellitus, chronic kidney disease, chronic obstructive pulmonary disease, and current antihypertensive medication use. In all cases of hypothesis testing, “No OH” was the reference group.

^a^ Geometric mean (standard error); all other values are the arithmetic mean (standard error).

* Statistically significant after Bonferroni adjustment (*P* < .0036).

In multiple logistic regression analyses, we calculated the odds of consensus OH, and its components, with elevated levels of phosphate, IL‐6, VWF, D‐dimer, hs‐troponin T, and NT‐proBNP (levels of biomarkers were categories into tertiles and “elevated” was defined as top tertile vs bottom) (Table 6). Elevated phosphate was statistically significantly associated with consensus OH and isolated diastolic OH; elevated VWF with consensus OH and isolated systolic OH; elevated hs‐troponin T with isolated diastolic OH and elevated NT‐proBNP with combined systolic and diastolic OH. Elevated IL‐6 appeared to be associated with combined systolic and diastolic OH, as did elevated D‐dimer, but these associations were not statistically significant.

**Table 6:** Odds of consensus OH, and its components, when levels of cardiovascular risk markers are elevated (the markers were grouped into tertiles and “elevated” was defined as top tertile vs bottom tertile)

	No OH (n = 3079)	COH (n = 778)	ISOH (n = 485)	IDOH (n = 177)	SDOH (n = 116)
	Odds ratio (95% Confidence interval)				
Phosphate, mmol/L	1.00	1.39 (1.13‐1.71)	1.22 (0.95‐1.57)	1.53 (1.04‐2.25)	2.12 (1.31‐3.44)
IL‐6, pg/mL	1.00	1.05 (0.84‐1.31)	0.97 (0.75‐1.27)	1.04 (0.68‐1.57)	1.51 (0.89‐2.55)
VWF, IU/dL	1.00	1.45 (1.18‐1.79)	1.35 (1.05‐1.73)	1.38 (0.92‐2.08)	2.27 (1.35‐3.83)
D‐dimer, ng/mL	1.00	1.08 (0.86‐1.35)	0.99 (0.75‐1.30)	1.22 (0.80‐1.86)	1.34 (0.79‐2.28)
NT‐proBNP, pg/mL	1.00	1.13 (0.88‐1.45)	0.97 (0.72‐1.30)	1.16 (0.72‐1.88)	2.14 (1.14‐4.03)
Hs‐troponin T, pg/mL	1.00	1.10 (0.88‐1.38)	0.99 (0.76‐1.30)	1.69 (1.07‐2.65)	0.87 (0.51‐1.50)

Abbreviations: COH, consensus orthostatic hypotension; IDOH, isolated diastolic orthostatic hypotension; ISOH, isolated systolic orthostatic hypotension; SDOH, combined systolic and diastolic orthostatic hypotension.

The cut‐offs for individual tertiles were as follows: Phosphate (mmol/L) tertile 1 (T1) < 1.10, tertile 2 (T2) ≥ 1.10 to <1.22, tertile 3 (T3) ≥ 1.22; VWF (IU/dL) T1 < 116, T2 ≥ 116 to <155, T3 ≥ 155, D‐dimer (ng/mL) T1 < 58, T2 ≥ 58 to <104, T3 ≥ 104; NT‐proBNP (pg/mL) T1 < 59, T2 ≥ 59 to <140, T3 ≥ 140, hs‐troponin T (pg/mL) T1 < 9.8, T2 ≥ 9.8 to <14.3, T3 ≥ 14.3. The odds ratios were calculated from multiple logistic regression models, which adjusted for age, body mass index, mean sitting systolic blood pressure, resting pulse, total cholesterol, smoking status, alcohol consumption, physical activity, social class and the presence of prevalent stroke, myocardial infarction, atrial fibrillation, diabetes mellitus, chronic kidney disease, and current antihypertensive medication use. In all cases, “No OH” was the reference group for hypothesis testing.

## 4 DISCUSSION

In this study of older, community‐dwelling British men, consensus OH was common. The vast majority of men with consensus OH did not report symptoms of breathlessness or feeling faint on standing. Of the components of consensus OH, isolated systolic OH was most common; it was almost three times as prevalent as isolated diastolic OH. Men with consensus OH had elevated levels of blood markers that are implicated in endothelial dysfunction, vascular calcification, myocardial injury, and cardiac stress, compared to men without consensus OH. Our findings are consistent with a previous study reporting OH to be associated with a more adverse cardiovascular profile.^9^

Men with consensus OH were older, had higher blood pressure and resting heart rate, compared to those without consensus OH. Although smoking and alcohol consumption are known to affect blood pressure,^36^, ^37^ there was no statistically significant difference in prevalence of consensus OH among men who were current smokers and those who reported moderate to heavy alcohol consumption. Possible explanations for this include misclassification of men with and without OH (repeated measurements of orthostatic blood pressure across a period of time (days or weeks) may have permitted more accurate classification) and/or the possibility that smoking and alcohol consumption may only affect postural blood pressure measurements in the acute period following consumption.

Orthostatic hypotension has been shown to increase risk of cardiovascular disease, including stroke, heart failure, atrial fibrillation, and myocardial infarction.^4^, ^5^, ^6^, ^7^ Our findings highlight the association between specific circulating cardiovascular risk markers and OH, providing further evidence that OH may be associated with increased cardiovascular risk. Our study extends previous research by investigating a wider range of cardiovascular risk markers in an older cohort—in whom the burden of OH is greatest—and contrasting the risk profile of isolated systolic OH, isolated diastolic OH, and combined systolic and diastolic OH; a distinction that has, thus far, been made infrequently. The different components of OH may be of different prognostic value, with respect to risk of cardiovascular events, falls and cognitive function,^23^, ^38^, ^39^, ^40^ and the present findings, which show differing relationships between the components of OH and markers of cardiovascular risk, may help explain these differences.

### 4.1 OH, endothelial dysfunction, hemostatis, vascular calcification, and inflammation

Consensus OH was associated with elevated levels of VWF and phosphate. VWF is a glycoprotein involved in hemostasis and circulating levels may reflect both endothelial dysfunction or activation.^13^ In a small study of 178 patients with unexplained syncope, of whom only 49 had OH, elevated VWF was associated with OH^12^; the present study extends these findings to an older, larger and predominantly asymptomatic cohort of men. Orthostatic stress may trigger the coagulation system by fluid shift and increased intravascular pressures, resulting in endothelial cell activation, as reflected by increased VWF.^41^ Increased concentrations and activity of VWF may result in endothelial damage, platelet adhesion, and aggregation (thus, hypercoagulability), which may mediate atherogenesis.^12^ Our finding that D‐dimer—a marker of activated coagulation—is elevated in men with combined systolic and diastolic OH further supports the notion that OH may be associated with hypercoagulability, but the association between D‐dimer and OH was not statistically significant after Bonferroni adjustment. We are not aware of findings that have previously associated D‐dimer and OH.

Phosphate was associated with consensus OH, isolated diastolic OH, and combined systolic and diastolic OH. Phosphorus, found in the body as phosphate, is an essential mineral. Raised levels of phosphate are associated with increased risk of cardiovascular disease.^42^ We are not aware of phosphate previously being associated with OH. However, given that elevated serum phosphate may contribute to vascular calcification^43^, ^44^ and that hyperphosphatemia, in vitro, has been shown to cause endothelial cell apoptosis, which may impair endothelial integrity,^45^ it is biologically plausible that phosphate may contribute to OH, through detrimental effects on vascular function. Although the present analysis was cross‐sectional, and we are unable to comment on temporality based on our results alone, recent prospective data in a large cohort have shown OH to be associated with carotid intimal thickness and carotid plaque.^9^ These are features of atherosclerotic vascular disease, and vascular calcification is widely used as an indicator of atherosclerosis.^46^ Thus, our finding that elevated phosphate is associated with OH is consistent with the notion that OH is associated with subclinical cardiovascular disease.

C‐reactive protein is an inflammatory acute phase reactant and IL‐6 a pro‐inflammatory cytokine; both are associated with future risk of cardiovascular disease.^47^ We did not find an association between CRP and consensus OH, confirming previous findings.^48^ IL‐6 was associated with consensus OH in a univariate analysis, but this association was not statistically significant after adjustment for potential confounders, such as age, body mass index, and co‐morbid conditions. In a recent, small cross‐sectional study of 279 adults referred to a tertiary syncope unit, there was an association between OH and the inflammatory biomarkers midkine, immunoglobulin‐like transcript 3, and regenerating islet‐derived protein 4.^11^ Thus, the potential association between OH and inflammatory biomarkers needs to be examined further, particularly because inflammatory processes are strongly associated with cardiovascular disease.^49^ Further research may clarify whether only particular inflammatory mediators are associated with OH or whether the increased risk of cardiovascular disease that is associated with OH is independent of inflammatory processes.

### 4.2 OH, myocardial injury, and cardiac stress

Elevated hs‐troponin T, a marker of myocardial injury, was associated with isolated diastolic OH, while elevated NT‐proBNP, a marker of cardiac stress, was associated with combined systolic and diastolic OH. These findings may help explain longitudinal findings that have (a) associated the diastolic component of OH, but not the systolic component, with increased risk of myocardial infarction^23^ and (b) associated OH with incident heart failure.^4^ Most coronary blood flow occurs during diastole. Repeated decreases in diastolic blood pressure, as would be expected in people with diastolic OH, may result in intermittent episodes of myocardial hypoperfusion,^23^ leading to myocardial injury, and accounting for the elevated levels of hs‐troponin T in these people. Longer‐term, compensatory structural changes to the heart may arise as a result of this increased ischemic burden, and subsequent injury, that may be occurring on a microvascular level, predisposing to heart failure.

In the Atherosclerosis Risk in Communities Study, detectable hs‐troponin T was associated with systolic OH, but not diastolic OH, while isolated systolic OH and isolated diastolic OH were both associated with elevated NT‐proBNP.^9^ This is in contrast to the present study in which hs‐troponin T was more closely associated with the diastolic component of OH, rather than the systolic component, and NT‐proBNP was more strongly associated with combined systolic and diastolic OH. These differences may reflect age differences in the two cohorts, as participants in the Atherosclerosis Risk in Communities Study cohort were, on average, 15 years younger than that those in the present study.

### 4.3 Strengths and limitations

Strengths of our study include the wide range of biomarkers studied and that we examined the components of OH. However, our cohort consisted exclusively of men who were almost entirely of white European ethnicity, limiting the external validity of our findings. Although we examined the components of OH, the sample in each group was relatively small, raising the possibility of type II errors; the confidence intervals in Table 6 provide insights into the strengths of associations which could not be excluded. Likewise, although we adjusted for multiple statistical comparisons using the Bonferroni method, this conservative adjustment method may have also predisposed to type II errors. Furthermore, there were multiple differences between several baseline characteristics among people with and without OH and we are unable to exclude the possibility of residual confounding.

We did not have supine and standing measurements of blood pressure and instead used sitting and standing measurements to define OH as per thresholds that are usually applied to supine and standing measurements. Although supine‐to‐standing measurements are more frequently employed in research settings, sitting‐to‐standing measurements are often used in clinical practice for convenience. The decrease in blood pressure that occurs when changing position from supine to standing can be expected to be greater than that which occurs when changing position from sitting to standing because the shift in fluid is greater in the first scenario. It is unknown whether lower thresholds should be used to diagnose OH based on sitting‐to‐standing blood pressure measurements, compared to supine‐to‐standing measurements. If this were the case, then it is likely that our measures of risk are underestimates, as our study would have misclassified people who truly had OH as not having it. The lack of supine blood pressure measurements means we are unable to exclude supine hypertension as a confounding factor. Furthermore, we did not have blood pressure measurements beyond three minutes of standing and would have misclassified men who developed OH after a prolonged period of standing. It has previously been suggested that specific orthostatic changes in heart rate, if they accompany OH, can provide insight into whether the cause of OH is neurogenic or non‐neurogenic.^50^ Based on our findings alone, which do not account for orthostatic changes in heart rate, we are unable to comment on whether the associations of some markers of cardiovascular risk may differ between different causes of OH.

Finally, this analysis was cross‐sectional. We are unable to comment on temporal relationships: While OH appears to be associated with some markers of cardiovascular risk, we are unable to conclude definitively whether OH is simply a biomarker of vascular risk and cardiac dysfunction or if OH induces endothelial dysfunction, vascular calcification, myocardial injury, and/or cardiac stress.

## 5 CONCLUSIONS

In older men, OH is associated with some cardiovascular risk markers implicated in endothelial dysfunction, vascular calcification, myocardial injury, and cardiac stress. The markers are differentially distributed among the components of OH, suggesting the components may have different etiologies. Our findings support to the hypothesis that OH is associated with increased cardiovascular risk. They may help explain findings from observational studies that have shown OH to be associated with increased risk of cardiovascular disease, including stroke, myocardial infarction, and heart failure. They support the notion that patients with OH should have their cardiovascular risk assessed. Further, prospective work should continue to examine the components of OH and control for markers of cardiovascular risk when attempting to delineate a temporal relationship between OH and cardiovascular morbidity.

## CONFLICT OF INTEREST

None declared.

## AUTHOR CONTRIBUTIONS

AG involved in conceptualization, methodology, formal analysis, investigation, writing‐original draft, and visualization. SR and SPJ involved in writing‐review and editing. OP involved in formal analysis, data curation, and data validation. LL involved in data curation, project administration, and resources. PW involved in data curation, methodology, investigation, resources, writing‐review and editing. GW involved in conceptualization, methodology, formal analysis, investigation, resources, writing‐review and editing, and visualization.
